# Commonality and Specificity of Acupuncture Point Selections

**DOI:** 10.1155/2020/2948292

**Published:** 2020-07-27

**Authors:** Ye-Seul Lee, Yeonhee Ryu, Da-Eun Yoon, Cheol-Han Kim, Geesoo Hong, Ye-Chae Hwang, Younbyoung Chae

**Affiliations:** ^1^Department of Anatomy and Acupoint, College of Korean Medicine, Gachon University, Seongnam, Republic of Korea; ^2^KM Fundamental Research Division, Korea Institute of Oriental Medicine, Daejeon, Republic of Korea; ^3^Acupuncture & Meridian Science Research Center, College of Korean Medicine, Kyung Hee University, Seoul, Republic of Korea

## Abstract

**Objective:**

Because individual acupoints have a wide variety of indications, it is difficult to accurately identify the associations between acupoints and specific diseases. Thus, the present study aimed at revealing the commonality and specificity of acupoint selections using virtual medical diagnoses based on several cases.

**Methods:**

Eighty currently practicing Korean Medicine doctors were asked to prescribe acupoints for virtual acupuncture treatment after being presented with medical information extracted from 10 case reports. The acupoints prescribed for each case were quantified; the data were normalised and compared among the 10 cases using *z*-scores. A hierarchical cluster analysis was conducted to categorise diseases treated based on the acupoint prescription patterns. Additionally, network analyses were performed on the acupoint prescriptions, at the individual case and cluster level.

**Results:**

Acupoints ST36, LI4, and LR3 were most commonly prescribed across all diseases. Regarding the specific acupoints prescribed in each cluster, acupoints around the disease site (knee and lower back) were frequently used in cluster A (musculoskeletal symptoms), acupoints LI4, LR3, PC6, and KI3 were frequently used in cluster B (psychiatric symptoms), and acupoints ST36, LI4, LR3, PC6, CV12, and SP6 were frequently used in cluster C (several symptoms of diseases of internal medicine).

**Conclusions:**

The present study identified the commonality and specificity of acupoint selections based on virtual acupuncture treatments prescribed by practicing clinicians. Acupoint selection patterns, which were defined using a top-down approach in previous studies and classical medical texts, may be further elucidated using a bottom-up approach based on patient medical records.

## 1. Introduction

In the practice of acupuncture, it is important to select appropriate acupoints for the treatment of patients with various symptoms and/or diseases. A series of acupuncture studies were conducted to identify acupoint indications using data mining methods [1–3], but each individual acupoint is associated with a wide variety of indications and different diseases can be treated with different combinations of acupoints [4, 5]. Additionally, there are different acupoint distribution patterns among diseases [6]. Thus, it is difficult to account for the complex relationships among diseases and acupoint selections using direct one-to-one matching methods. In clinical practice, several primary acupoints, including LI4, LR3, and ST36, are widely applied across many different conditions, whereas other acupoints are used only for specific indications [5]. This core set of acupoints can be used to treat a variety of diseases; there is a wide range of acupoint indications but a relative lack of specificity. On the other hand, some acupoints are used to treat specific diseases. To effectively investigate the factors associated with accurate acupoint indications, an understanding of the commonality and specificity of each acupoint selections is necessary.

An emerging literature has described the variability inherent to acupoint selection in clinical practice [7]. For example, many different acupoints were selected for treating a sample of patients with frequent headaches [7], and acupuncture treatment recommendations for patients with chronic low back pain vary widely across practitioners [8]. Although there is substantial variability among acupuncture practitioners in terms of acupoint selection, core points for certain diseases still exist [5]. Different acupuncture practitioners tend to select different acupoints, but five or six core acupoints are used in more than 50% of cases [9]. Notably, the core acupoints differ among diseases; thus, the specificity of an acupoint indication can be estimated based on which acupoints are actually used to treat a certain disease in clinical practice.

In the present study, acupuncture practitioners were asked to participate in a virtual diagnosis process, a method employed in previous studies to evaluate the performance of the doctors and medical students [10–12]. In this study, we provided the cases with medical information extracted from 10 different case reports; acupuncture prescriptions were then collected from the virtual diagnosis data completed by the participating acupuncture practitioners. Using network analysis and hierarchical clustering methods, the commonality and specificity of various acupoint selections were assessed based on virtual acupuncture treatment prescriptions.

## 2. Methods

### 2.1. Experimental Design

The present study was an online experiment that assessed the virtual medical diagnoses provided by 80 Korean Medicine doctors for 10 different case reports. The baseline characteristics of the participating doctors are shown in Table 1. All participating doctors received a detailed explanation of the study and provided written informed consent prior to participation; the anonymity of the doctors was maintained throughout the study. All procedures were conducted with the approval of the Institutional Review Board of Kyung Hee University, Seoul, Republic of Korea (KHSIRB-17-046). The associations between the pattern identifications and selected acupoints have been described in detail in our previous study [13].

The doctors were asked to prescribe combinations of acupoints for each case, which were modified from the excerpts of actual case reports published in Korean medical journals between 2014 and 2016. The cases presented in the experiment included medical information such as major symptoms, medical history, and laboratory test results (Table 2).

### 2.2. Data Collection and Preprocessing

A total of 80 data per case were collected, i.e., 800 virtual diagnoses in total. For each case, the acupoints prescribed were recorded in a .txt format. Because the acupoint prescription data were recorded in a free-text format without any restrictions, the data required preprocessing prior to analysis to remove typographical errors and unify the different terminologies used for the acupoints. In the final data analysis, standard World Health Organization terminology was used for acupoints in the 14 meridians and any additional acupoints. Following this data conversion process, the free-text data were converted into the .csv file format and data preprocessing was conducted using an in-house script (“Short Summer”) in the R software environment (ver. 3.4.2; R Development Core Team, Vienna, Austria).

### 2.3. Probability Analysis of the Prescribed Acupoints

Using the preprocessed data, an acupoint selection probability (*P*) was calculated, as the count of each acupoint in each case divided by the total number of prescribed acupoints among the 30 most acupoints. For example, the frequency of ST36 in Case 1 was 35 and the total sum of prescribed acupoints among the 30 most frequently prescribed acupoints was 232, and the probability of ST36 in Case 1 was 0.151 (=35/232). Next, the data were normalised and the *z*-scores of the acupoints were obtained using the mean and standard deviation of the frequency of each acupoint among the 30 most acupoints, to calculate how many standard deviations each acupoint was from the mean and place the probability of each acupoint on a normal distribution curve. Both the probability and *z*-scores of the 30 most commonly prescribed acupoints of the 10 cases were illustrated on heatmaps. These procedures were conducted using an in-house script (“Short Summer”) and the “plot3D” package in R software.

### 2.4. Hierarchical Clustering Analysis of the Prescribed Acupoints

Using the normalised probabilities (i.e., *z*-scores) of the prescribed acupoints, the 10 cases in the present study were clustered using a hierarchical clustering analysis with Ward's minimum variance criterion. The hierarchical cluster analysis technique is often used for data mining and statistics, to partition objects into optimally homogeneous groups based on measures of similarity [14]. Of the various hierarchical clustering analysis methods, Ward's minimum variance criterion is an agglomerative approach that minimises total within-cluster variance [15]. In the present study, the hierarchical clusters based on the prescribed acupoints in each case were then illustrated in a dendrogram that showed the final three clusters. These procedures were conducted using an in-house script (“Short Summer”) in R software. Additionally, frequently prescribed acupoints in each cluster were imposed on an image of the human body.

### 2.5. Network Analysis of the Combinations of Acupoints

To analyse how different acupoint combinations were prescribed in different cases, an undirected network was constructed with edges that connected the acupoints. The acupoint prescriptions for a single case, made by a single doctor, were considered to be one set and the acupoints within that set were linked. One prescription was given a weight of 1, which was shared between the links that connected the acupoints within the prescription. Consequently, the weight given to each link within one prescription was calculated as a reciprocal of *C*_(*n*, 2)_, where *n* is the number of acupoints. For example, if three acupoints were prescribed in one case by a single doctor, each of the links between the acupoints had a weight of 0.33. The edges that formed the undirected network were constructed using the sum of the weights between two acupoints across all cases.

Edges that had weights greater than the mean were selected for the final network. Then, the eigenvector centrality values of the acupoints were computed and illustrated using a colour scale, such that acupoints with greater centrality were darker; the weight was illustrated by the width of the edges. Lastly, acupoint networks of the aforementioned clusters were constructed to examine prescription patterns according to cluster. All three networks were computed separately to independently calculate the weight and eigenvector centrality values, which allowed for visualisation of frequently prescribed acupoints in different clusters. All network analyses were computed with the “igraph” package in R and visualised using Gephi software (ver. 0.9.3).

## 3. Results

### 3.1. Probabilities of the Prescribed Acupoints on Each Disease

**Figure 1:**
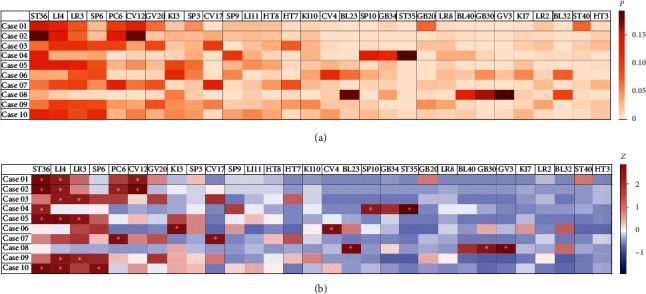
(a) Probabilities of each acupoint in the 10 cases. Acupoint probability was calculated as the frequency of the acupoint prescribed for the designated case divided by the total number of prescribed acupoints among the 30 most acupoints. (b) The *z*-score heatmap for each acupoint in the 10 cases. The *z*-score was calculated by subtracting the probability of the acupoint prescribed for the designated case from the mean of the prescribed acupoints for all cases and then dividing by the standard deviation of the probability of prescribed acupoints for all cases. The 30 most frequently prescribed acupoints are on the *x*-axis and the 10 cases are on the *y*-axis. ^*∗*^represents *Z* > 1.96.

The 30 most frequently prescribed acupoints are listed in descending order of their probability values for the 10 cases (Figure 1). Among all cases, the most frequently prescribed acupoints were ST36 (*P*=0.100), LI4 (*P*=0.091), LR3 (*P*=0.078), SP6 (*P*=0.061), PC6 (*P*=0.057), and CV12 (*P*=0.056) (chance level = 0.033). The probability values of the 30 acupoints for the 10 cases are also shown on a heatmap (Figure 1(a)).

The *z*-scores illustrate how frequently each acupoint was prescribed in each case compared to the overall mean prescription frequency for that case. The *z*-scores of the 30 acupoints for the 10 cases are depicted on a heatmap (Figure 1(b). Acupoints with a high *z*-score (*Z* > 1.96) are as follows: ST36 (*Z* = 2.65), LI4 (*Z* = 2.07), and CV12 (*Z* = 2.17) in Case 1 (vertigo); ST36 (*Z* = 2.79), LI4 (*Z* = 2.10), PC6 (*Z* = 2.02), and CV12 (*Z* = 2.64) in Case 2 (gastroesophageal reflux disease); L14 (*Z* = 2.05) and LR3 (*Z* = 2.05) in Case 3 (menopausal climacteric states); ST36 (*Z* = 1.98), SP10 (*Z* = 2.07), and ST35 (*Z* = 3.11) in Case 4 (acute traumatic meniscal tears); ST36 (*Z* = 2.53), LI4 (*Z* = 2.53), and LR3 (*Z* = 2.24) in Case 5 (diabetic neuropathy); KI3 (*Z* = 2.50) and CV4 (*Z* = 2.35) in Case 6 (chronic prostatitis); PC6 (*Z* = 2.43) and CV17 (*Z* = 2.43) in Case 7 (panic disorder); BL23 (*Z* = 2.57), GB30 (*Z* = 2.20), and GV3 (*Z* = 2.81) in Case 8 (lumbar herniated intervertebral disc); L14 (*Z* = 2.30) and LR3 (*Z* = 2.00) in Case 9 (fibromyalgia); and ST36 (*Z* = 2.66), LI4 (*Z* = 2.33), and SP6 (*Z* = 2.33) in Case 10 (hyperhidrosis and joint pain).

### 3.2. Hierarchical Clusters of the Prescribed Acupoints

**Figure 2:**
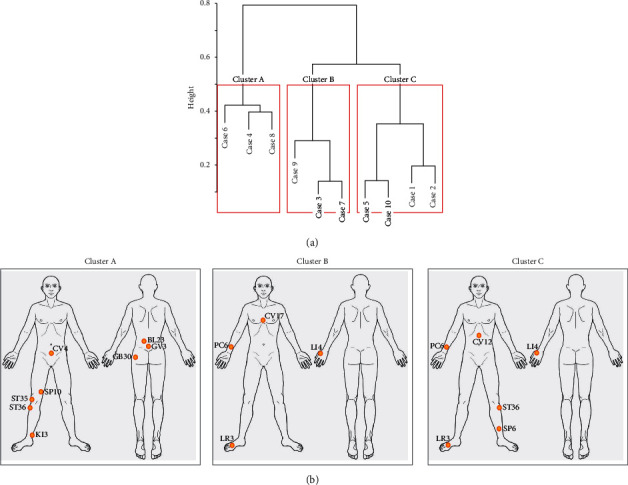
(a) Hierarchical clustering dendrogram. The red box shows the final clusters generated by the analysis; the clusters are labelled as cluster A, cluster B, and cluster C. The *y*-axis shows the squared Euclidean distance between cluster centres. (b) Specific acupoints within each cluster imposed on an image of a human body.

Figure 2(a) shows a dendrogram of the hierarchical clusters of cases. Using Ward's method, the cases with minimum variance among the normalised probability of the acupoints were clustered; the *y*-axis of the dendrogram refers to the squared Euclidean distance between cluster centres. Based on the acupoint prescriptions, three clusters of cases were defined in the clustering analysis: Cluster A included Cases 4, 6, and 8; Cluster B included Cases 3, 7, and 9; and Cluster C included Cases 1, 2, 5, and 10. Cluster A included cases with musculoskeletal symptoms, such as knee pain, pelvic pain, and back pain; Cluster B included cases with psychiatric symptoms, such as depression, panic, and insomnia; and Cluster C included cases with various symptoms treated in internal medicine, such as dizziness, heartburn, cold hands and feet, and hyperhidrosis (Table 2).

### 3.3. Disease-Specific Acupoint Indications for Each Cluster

Figure 2(b) shows the acupoints that frequently occurred in each cluster, imposed on an image of a human body. For musculoskeletal diseases (Cluster A), ST36, SP10, and ST35 were used to treat acute traumatic meniscal tears, KI3 and CV4 were used to treat chronic prostatitis with pelvic pain, and BL23, GB30, and GV3 were used to treat cases of a lumbar herniated intervertebral disc. For psychiatric disorders (Cluster B), LI4 and LR3 were used to treat menopausal climacteric states and fibromyalgia with depression and PC6 and CV17 were frequently used to treat panic disorder. For diseases of internal medicine (Cluster C), ST36 and LI4 were highly associated with vertigo, gastroesophageal reflux disease, diabetic neuropathy, and puerperal disorder and CV12 was used to treat vertigo and gastroesophageal reflux disease. PC6 was used to treat gastroesophageal reflux disease, LR3 was used to treat diabetic neuropathy, and SP6 was used to treat puerperal disorder.

### 3.4. Network Analysis of the Prescribed Acupoints

**Figure 3:**
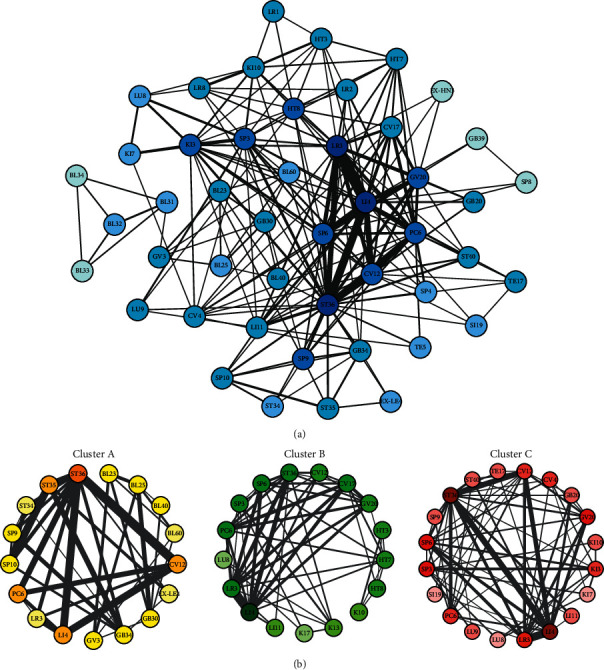
(a) Network analysis of acupoint prescriptions among all cases. The eigenvector centrality values of the acupoints are illustrated on a colour scale and the weights of the links between the acupoints correspond to the width of the edges. (b) Acupoint networks according to hierarchical cluster analysis. The acupoint patterns in each cluster are illustrated in different colours. The eigenvector centrality values of the acupoints are illustrated on a colour scale, and the weights of the links between the acupoints correspond to the width of the edges.

The final network that encompassed all cases included a total of 47 acupoints. The acupoints with the highest eigenvector centrality values were ST36 (1.00), LI4 (0.99), LR3 (0.98), SP6 (0.82), and PC6 (0.79), which indicates that these acupoints had the most connections with other acupoints (denoted by the width of the edges in Figure 3(a)). In order, the acupoint pairs with the highest weights were LI4-LR3, LI4-ST36, ST36-CV12, and LI4-CV12 (Table 3).

The networks of the three clusters that show the prescribed acupoints in each cluster are illustrated in Figure 3(b). In Cluster A, only acupoints located around the knee (ST34, ST35, GB34, SP10, EX-LE4, and BL40) and at the back (BL23 and BL25) and ankle (BL60) were prescribed. In Cluster B, only CV17 and HT7 were prescribed. In Cluster C, only GB20, LU9, and SI19 were prescribed. The acupoints with the highest eigenvector centrality values, such as ST36, LI4, and LR3, were frequently included in all three clusters and have darker colours and thicker edges on their connections.

## 4. Discussion

The present study evaluated the commonality and specificity of acupoints prescribed for 10 different cases using a series of statistical approaches. Eighty currently practicing Korean acupuncture clinicians made virtual diagnoses. A frequency analysis revealed acupoints that were commonly used across all cases, and *z*-score distributions showed the acupoints that were prescribed in each individual case. Hierarchical clustering analyses that defined clusters of cases according to the pattern of prescribed acupoints revealed three primary clusters: musculoskeletal symptoms, psychiatric symptoms, and symptoms treated in internal medicine. A network analysis revealed the interconnected acupoints, as well as differences in acupoint prescription patterns among the case clusters. The present findings showed the acupoints that were commonly prescribed across different cases, including combinations thereof. Previous studies have shown the interrater reliability of traditional Chinese medicine (TCM) diagnosis of a variety of diseases including prediabetes [16], cardiovascular disease [17], rheumatoid arthritis [18], and irritable bowel syndrome [19]. Further studies showed the difficulties in standardizing the TCM pattern diagnosis and the importance of collecting medical data [20, 21]. This study further extends the previous studies in the work of collecting and standardizing traditional medicine diagnosis and treatment prescriptions by providing a larger set of data and applying data mining technique.

According to the analysis of acupoint prescriptions among all cases, the acupoints with the highest probabilities were ST36, LI4, and LR3. These are the most important and commonly used acupoints for a variety of symptoms and have been studied extensively [7, 22]. The *z*-score analyses in the present study revealed that some acupoints were statistically more frequently prescribed in certain cases. For example, CV12 had a high *z*-score in Case 2, which can be explained by reference to previous studies showing that CV12 is commonly prescribed to treat heartburn [23] and gastroesophageal reflux disease [24]. On the other hand, BL23, GB30, and GV3, which are all located around the back and hips, had high *z*-scores in Case 8, which was characterised by primary symptoms of low back pain.

The use of a hierarchical clustering algorithm enabled the grouping of cases according to their acupoint prescription patterns. Three clusters were defined in this analysis: Cluster A included Cases 4, 6, and 8, which were characterised by musculoskeletal symptoms such as knee pain, pelvic pain, and back pain as their major symptoms; Cluster B included Cases 3, 7, and 9, which were characterised by mental illnesses such as depression, panic disorder, and sleeping disorder as their major symptoms; and Cluster C included Cases 1, 2, 5, and 10, which were characterised by symptoms treated in internal medicine, such as dizziness, heartburn, cold hands and feet, and hyperhidrosis. Through an agglomerative approach, this analysis revealed that acupoint prescription patterns among cases with similar symptoms were similar to each other, but distinct from those of other cases.

Network analyses revealed the most commonly prescribed acupoints in the present study. The acupoints that had the highest eigenvector centrality values, ST36, LI4, and LR3, and the acupoint pairs with the highest weights overlapped with those with the highest probabilities of the prescibed acupoints. Furthermore, within the network, these common acupoints were linked to other acupoints that were specific to certain other cases. The networks created by the hierarchical clusters illustrate in detail how common and specific acupoints were used in combination. Although ST36, LI4, and LR3 were included in all three clusters, they were prescribed in combination with acupoints that were specific to each cluster. In Cluster A, ST36 was linked to nearby acupoints located around the knee, such as ST34, ST35, GB34, SP10, EX-LE4, and BL40. Additionally, bladder meridian (BL) acupoints located at the back, such as BL23 and BL25, and at the ankle, such as BL60, were prescribed only in this cluster. Meanwhile, ST36 was linked to CV17 and HT7 in Cluster B and appeared only in this cluster. In Cluster C, ST36 was connected to GB20 and LU9, which were acupoints specific to this cluster.

Examined more closely, the specific acupoints in each cluster that were prescribed in conjunction with the common acupoints together represent the main symptoms of the cluster. In Cluster A (musculoskeletal symptoms), acupoint prescriptions were based on local acupoints near the knee or back. The abovementioned prescribed acupoints have been assessed in previous studies that investigated the effectiveness of acupuncture for musculoskeletal diseases such as knee osteoarthritis [25] and lumbar disc herniation [26]. Acupoints around the knee and the lower back such as ST36 have been reported to be effective in osteoarthritis and disc herniation by alleviating inflammation and regulating immune activity [27–29]. In Cluster B (psychiatric symptoms), CV17 and HT7 were the specific acupoints. Traditional East Asian Medicine theories suggest that HT7 are effective in psychiatric symptoms due to the heart meridian's nature to regulate emotions [30]. Previous studies have shown that CV17 is used to treat Hwa-byung and anxiety [31, 32], while HT7 is used to treat sleep disorders [33–35] and depression-related behaviours [36–38]. Furthermore, HT7 and GV20 are the most frequently suggested acupoints for treating mental diseases according to the National Standard of Acupoint Indications in China [39]. In Cluster C in this study, GB20 and LU9 were the specific acupoints. Previous studies have shown that GB20 is used to treat vertigo [40, 41], as well as in the traditional medical literatures which indicate that gallbladder meridian treats vertigo and dizziness [42]. On the other hand, LU9 is prescribed for treating dry eye [43] and chronic skin problems throughout the body, including atopic dermatitis [44] and psoriasis [45]. Interestingly, previous studies explained that theories in traditional Korean Medicine relate skin problems to lung dryness [45].

Based on the virtual diagnoses of currently practicing doctors, the present results suggest a relationship between symptom indications and acupoint prescriptions in Korean Medicine. The selected cases varied in terms of disease classification and major symptoms, to maximise the variability of acupoint selections and ensure data were obtained for a wide range of diseases. Although there is substantial variability among doctors in terms of acupoint prescriptions, there are also core acupoints for specific diseases [5]. The present study extended current findings by obtaining virtual diagnoses of various cases, where the most frequently prescribed acupoints were identified and examined for commonalities and specificities. The results revealed clear patterns of acupoints commonly prescribed across various diseases, as well as acupoints used in specific cases. Additionally, the present cases could be clustered based on acupoint prescription patterns, i.e., musculoskeletal, psychiatric, or diseases in internal medicine. The specific acupoints relevant to each of these clusters have been identified in previous studies and can be effective for addressing the symptoms associated with these clusters. Whereas previous studies employed a top-down approach based on symptom indications for acupoint selection, the present study examined acupoint prescription patterns using a bottom-up approach that was based on data generated by clinicians. The following definition of an acupoint indication is suggested: an acupoint indication is a set of symptoms and signs for which the selected acupoint has been prescribed and shown to be effective in clinical practice.

This definition of acupoint indications has been suggested in previous studies [1, 46] that constructed spatial patterns of acupoint indications using both classical medical texts and medical records. Additionally, a complex network analysis that used medical textbooks as data sources revealed an association between acupoints and symptoms [6]. The present findings improved our understanding of acupoint selection patterns according to virtual diagnoses made by currently practicing clinicians; a large set of acupoint selection data was generated. Using the acupoint selection data and the diagnosis records, we conducted additional studies on the relationship between pattern identification and acupoint selections which showed commonly applied and disease-specific acupoints [13]. There are two different approaches to acupoint prescription, one of which is based on the disease/symptoms and the other is based on the results of pattern identification [47]. It is noteworthy that both of these approaches can be identified using the data mining approach. This approach could also be applied to examine the effectiveness of acupoint indications and prescription patterns in real practice and to support acupuncture education and research. Data mining techniques and network analyses of medical data could help characterise acupoint indications [48, 49].

The present study had several limitations that should be considered. First, the effectiveness of acupuncture interventions could not be evaluated due to the nature of the experiment (virtual diagnoses). To fully understand the symptom indications for both the acupoints common among cases and those specific to individual cases, it will be necessary to review the clinical efficacy of each acupoint prescription pattern through other types of data including real-world medical records, randomized clinical trial data, and traditional medical texts. Second, the findings were based on data from 80 currently practicing doctors; while this sample size represents an increase from previous studies investigating acupoint indications based on medical records [1], prospective studies should include a larger database to characterise acupoint indications across a wider range of diseases and a comprehensive sample size, including practitioners from across the countries such as China and Japan, to cover a wide range of skills variations among the doctors. In addition, the present study utilised case reports to create vignettes; although the study was designed to include all patient information provided in the case reports, future studies including other types of cases, such as those described in classical medical texts and/or real patients in clinical settings, will be necessary. Finally, further study is needed to investigate the relationship between the acupoint selection patterns and pattern identification, which is the main diagnostic method employed by many Korean Medicine doctors.

In conclusion, the present study evaluated acupoint selection patterns based on virtual diagnoses. Statistical analyses and data mining revealed acupoints that were common among all cases, as well as those that were specifically prescribed in certain cases. Based on the acupoint prescription patterns generated by currently practicing clinicians, the cases could be grouped into clusters that exhibited similar symptoms. A network analysis revealed interconnections between acupoints, as well as differences in acupoint patterns among the clusters. Based on the commonalities and specificities of the examined acupoints, the present findings suggest that acupoint selection patterns, which to date have been defined using a top-down approach based on data from previous studies and classical medical texts, may be better explained by a bottom-up approach based on medical records.

## Figures and Tables

**Table 1:** Baseline characteristics of the participating doctors (*n* = 80).

Categories		Number of doctors (%)
Age	20–29	14 (17.5%)
	30–39	52 (65.0%)
	40–49	13 (16.3%)
	50+	1 (1.3%)

Gender	Male	52 (65.0%)
	Female	28 (35.0%)

Experience	1–4 years	33 (41.3%)
	5–9 years	32 (40.0%)
	10+ years	15 (18.8%)

Medical institution	Public medical clinics	16 (20.0%)
	Private clinics	35 (43.8%)
	Hospitals (Korean Medicine/Western Medicine)	26 (32.5%)
	Nursing hospitals	3 (3.8%)

Employed status	Paid by the institution	41 (51.3%)
	Runs an own institution	39 (48.8%)

Area of specialization	Musculoskeletal disorders	38 (47.5%)
	Gynecology and obstetrics	6 (7.5%)
	Otorhinolaryngology	3 (3.8%)
	Obesity	1 (1.3%)
	Dermatology	2 (2.5%)
	Oncology	1 (1.3%)
	Neurology	4 (5.0%)
	N/A	25 (31.3%)

Type of profession	General practitioner	31 (38.8%)
	Resident	9 (11.3%)
	Specialist	11 (13.8%)
	N/A	29 (36.3%)

Total		80

**Table 2:** Descriptions of the medical cases assessed in this study.

Case	Disease (ICD-10)	Main symptoms	Reference journal	Published year
1	Benign paroxysmal positional vertigo (H81.1)	Dizziness	Journal of Korean Oriental Internal Medicine	2016

2	Gastroesophageal reflux disease (K21.0)	Heartburn and nausea	Journal of Korean Oriental Internal Medicine	2015

3	Menopausal climacteric states (N95.1)	Forgetfulness, depression, muscle pain, heart palpitations	The Journal of Oriental Gynecology	2016

4	Derangement of meniscus (M23.2)	Knee pain	The Acupuncture	2015

5	Diabetic neuropathy (E10.4)	Cold and numb hands and feet	Journal of Korean Oriental Internal Medicine	2016

6	Chronic prostatitis (N41.1)	Pelvic pain and hip joint pain	The Acupuncture	2014

7	Panic disorder (F41.0)	Heart palpitations and chest tightness	Journal of Oriental Neuropsychiatry	2014

8	Intervertebral disc disorders (M51.0)	Low back pain, difficulty in walking	The Acupuncture	2015

9	Fibromyalgia (M79.7)	Burning sensation in the face, arms, and legs, insomnia, and depression	Journal of Korean Oriental Internal Medicine	2015

10	Puerperal disorder (U32.7)	Hyperhidrosis and joint pain throughout the body	The Journal of Oriental Gynecology	2015

**Table 3:** Acupoint pairs with the highest weights in the network analysis (undirected).

Rank	Acupoint 1			Acupoint 2			Weight
	Standard nomenclature	Pinyin	Korean	Standard nomenclature	Pinyin	Korean	
1	LI4	Hé gǔ	Hap gok	LR3	Taì chōng	Tae chung	37.7
2	LI4	Hé gǔ	Hap gok	ST36	Zú sān lǐ	Jok sam ni	26.5
3	ST36	Zú sān lǐ	Jok sam ni	CV12	Zhōng wǎn	Jung wan	21.8
4	LI4	Hé gǔ	Hap gok	CV12	Zhōng wǎn	Jung wan	18.3
5	LR3	Taì chōng	Tae chung	ST36	Zú sān lǐ	Jok sam ni	16.9
6	ST36	Zú sān lǐ	Jok sam ni	PC6	Nèi guān	Nae gwan	16.5
7	LI4	Hé gǔ	Hap gok	SP6	Sān yīn jiāo	Sam eum gyo	14.3
8	LR3	Taì chōng	Tae chung	SP6	Sān yīn jiāo	Sam eum gyo	12.6
9	LI4	Hé gǔ	Hap gok	GV20	Bǎi huì	Baek hoe	11.8
10	LR3	Taì chōng	Tae chung	CV12	Zhōng wǎn	Jung wan	11.6
11	LI4	Hé gǔ	Hap gok	PC6	Nèi guān	Nae gwan	11.4
12	ST36	Zú sān lǐ	Jok sam ni	SP6	Sān yīn jiāo	Sam eum gyo	11.2
13	LR3	Taì chōng	Tae chung	GV20	Bǎi huì	Baek hoe	10.5
14	CV12	Zhōng wǎn	Jung wan	PC6	Nèi guān	Nae gwan	10
15	KI3	Taì xī	Tae gye	SP3	Taì bái	Tae baek	9.8
16	PC6	Nèi guān	Nae gwan	CV17	Dàn zhōng	Dan jung	9.3
17	LR3	Taì chōng	Tae chung	PC6	Nèi guān	Nae gwan	8.9
18	ST36	Zú sān lǐ	Jok sam ni	LI11	Qū chí	Gok ji	8.4
19	LI4	Hé gǔ	Hap gok	CV17	Dàn zhōng	Dan jung	8.3
20	ST36	Zú sān lǐ	Jok sam ni	GV20	Bǎi huì	Baek hoe	8.2
21	CV17	Dàn zhōng	Dan jung	GV20	Bǎi huì	Baek hoe	7.6
22	PC6	Nèi guān	Nae gwan	GV20	Bǎi huì	Baek hoe	7.5
23	ST36	Zú sān lǐ	Jok sam ni	SP3	Taì bái	Tae baek	7.5
24	LR3	Taì chōng	Tae chung	CV17	Dàn zhōng	Dan jung	7.3
25	KI10	Yīn gǔ	Eum gok	LR8	Qū quán	Gok cheon	7.2
26	SP6	Sān yīn jiāo	Sam eum gyo	KI3	Taì xī	Tae gye	7
27	GV20	Bǎi huì	Baek hoe	CV12	Zhōng wǎn	Jung wan	7
28	ST36	Zú sān lǐ	Jok sam ni	SP9	Yīn líng qúan	Eum neung cheon	6.8
29	PC6	Nèi guān	Nae gwan	SP6	Sān yīn jiāo	Sam eum gyo	6.7
30	KI3	Taì xī	Tae gye	KI7	Fù liū	Bu ryu	6.7

## Data Availability

The data used to support the findings of this study are included within the article.
